# Green Synthesis of Copper Nanoparticles from the Aqueous Extract of *Lonicera japonica* Thunb and Evaluation of Its Catalytic Property and Cytotoxicity and Antimicrobial Activity

**DOI:** 10.3390/nano15020091

**Published:** 2025-01-09

**Authors:** Weijie Yu, Jingyi Tang, Chunxia Gao, Xuesong Zheng, Peizhi Zhu

**Affiliations:** 1 School of Chemistry and Chemical Engineering, Yangzhou University, Yangzhou 225002, China; mz120240673@stu.yzu.edu.cn (W.Y.); mz120220781@stu.yzu.edu.cn (J.T.); cxgao@yzu.edu.cn (C.G.); 2 School of Perfume and Aroma Technology, Shanghai Institute of Technology, Shanghai 201418, China

**Keywords:** green synthesis, *Lonicera japonica* Thunb, Cu NPs, dyes reduction, cytotoxicity, antibacterial

## Abstract

In this study, copper nanoparticles with an average particle size of 2–4 nm were synthesized using the green extract of *Lonicera japonica* Thunb. The catalytic activity and dye degradation efficiency of Cu NPs were evaluated using ultraviolet spectroscopy. To confirm that Cu NPs can continuously remove organic dyes, this study used Cu/Lj-C composite material adsorbed on cotton balls as a simulated bed to study the cyclic catalytic activity of Cu NPs for the reduction of methylene blue by sodium borohydride (NaBH_4_). The experiment showed that after multiple cycles, it can also quickly and effectively reduce methylene blue. To evaluate the toxicity of Cu NPs, experiments were conducted using HUVEC and MC3T3-E1 cells. The median lethal doses (LD_50_) were 37.64 µg/mL and 7.50 µg/mL. The synthesized Cu NPs also exhibited antibacterial efficacy against *Aspergillus niger* (fungus), *Staphylococcus aureus* (Gram-positive bacteria), *Escherichia coli* (Gram-negative bacteria), and *Candida albicans* (yeast).

## 1. Introduction

The textile industry utilizes over 10,000 different types of dyes, with approximately 700,000 metric tons of synthetic dyes produced annually [1,2]. It is estimated that 20% of these dyes are lost during the production and processing of textiles, significantly contributing to organic water pollution [3]. Moreover, organic dyes are known to possess genotoxic and mutagenic properties, with the potential to cause DNA damage, which can have detrimental effects on human health [4,5]. Fortunately, these dyes can be effectively treated through various methods, such as catalytic reduction, photocatalytic degradation, and adsorption [6,7]. The performance of catalysts can be improved by modifying reaction pathways or lowering energy barriers during the reaction process. Recent research has focused on the use of nanomaterials as catalysts in a variety of catalytic applications [8]. By manipulating the size and structure of nanomaterials during the nanocatalysis process, it is possible to produce catalysts with enhanced activity, selectivity, and stability [9,10,11]. Metal nanoparticles, including platinum, silver, gold, copper, and others, are commonly employed as nanocatalysts in catalytic reduction reactions [12]. The reason for choosing copper as the research object is that Cu NPs are easily obtainable, cost-effective, and possess similar chemical properties to metals such as silver and gold.

Polyphenols in plant extracts are highly oxidizable [13] and non-toxic, making them ideal reducing agents in green synthesis processes. Unlike traditional chemical and physical methods, green synthesis techniques use natural reactants such as algae, bacteria, and plants, providing a more cost-effective and environmentally friendly alternative [14,15,16]. The plant extract in an aqueous solution stabilizes nanoparticles, preventing oxidation and aggregation during the reduction process [17]. Plant-mediated green nanoparticle synthesis offers a promising approach for producing eco-friendly and functional copper nanoparticles suitable for various applications [18,19].

*Lonicera japonica* Thunb (*L. japonica*), a perennial, semi-evergreen plant from the Lonicera genus, is native to East Asia but has since spread to South America, North America, Hawaii, and Australia [20,21]. This plant possesses various pharmacological properties, including anti-inflammatory, antiviral, antioxidant, hepatoprotective, blood lipid-lowering, and blood glucose-lowering effects [22,23]. The flowers of *Lonicera japonica* contain chlorogenic acids, notably neochlorogenic acid (5-CQA) and isochlorogenic acid (3,5-diCQA) [24]. Copper nanoparticles (Cu NPs) have been synthesized using plant extracts rich in chlorogenic acid, including those from walnut shells [25], green coffee beans [26], Orobanche aegyptiaca extract [27], and aconitic acid [28]. The reason for choosing honeysuckle extract is that it contains a higher amount of chlorogenic acid and is affordable, offering economic benefits.

**Scheme 1:**
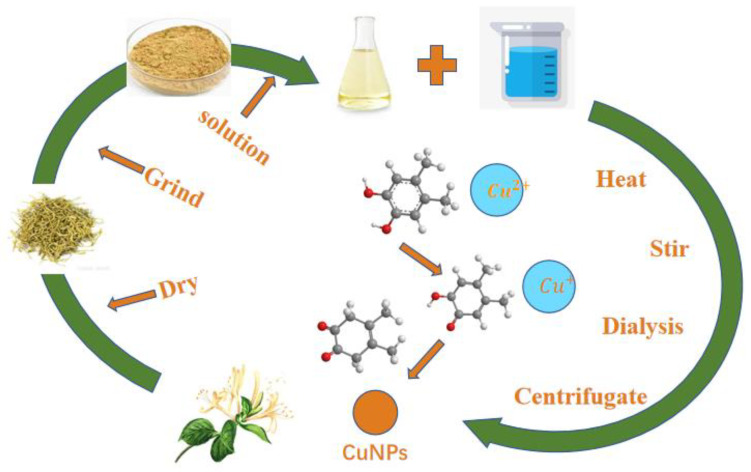
Synthesis of nano-copper by green method.

Nanoparticles pose potential hazards due to their high surface-to-volume ratio, reactivity, and interactions with biomolecules [29]. Therefore, understanding the ecotoxicological impact of green nanoparticles is crucial for environmental protection [30,31]. In this study, the aqueous extract of *Lonicera japonica* Thunb was selected as a reducing, capping, and stabilizing agent, as outlined in Scheme 1. The structure and morphology of the synthesized nanoparticles were characterized using High-Resolution Transmission Electron Microscopy (HRTEM), and FTIR analysis was conducted in the 4000~500 cm⁻^1^ range to obtain structural details. The catalytic activity and dye degradation efficiency of the copper nanoparticles were assessed using UV spectroscopy. A simple catalytic bed using Cu NP-modified fiber balls was simulated for the continuous flow removal of organic dyes. Furthermore, the cytotoxicity of the copper nanoparticles was evaluated to ensure their safe use in biological research and applications, and their antibacterial and antifungal properties were also assessed.

Due to the rise in infectious diseases and the emergence of antibiotic-resistant strains, metal nanoparticles have become widely utilized as alternative antibacterial agents [32] in various applications. Copper nanoparticles (Cu NPs) exhibit antibacterial activity against *Proteus vulgaris*, *Escherichia coli*, *Pseudomonas aeruginosa*, *Klebsiella pneumoniae*, and *Streptococcus* species [33,34]. These nanoparticles have been incorporated into plastics and textiles for antimicrobial purposes, and their antibacterial properties also make them valuable in wound dressings [35]. This article also investigates the antibacterial properties of Cu NPs against *E. coli*, *Staphylococcus aureus*, and *Candida albicans* plates, further confirming their potential application as antibacterial agents.

## 2. Materials and Methods

### 2.1. Chemicals and Materials

Carmine crimson (CR, AR), methylene blue (MB), amido black 10B (AB-10B), and copper chloride (CuCl_2_, 99.8%) were obtained from Sinopharm Chemical Reagent Co., Ltd. (Shanghai, China) as well as sodium borohydride (NaBH_4_, 98%). Purchases were made from Shan Xi TIANXINGJIAN Natural Bio-products Co., Ltd. (Xi’an, China) for *Lonicera japonica* Thunb extracts. *Lonicera japonica* Thunb extract does not require processing and can be used directly. The substances were all of the analytical reagent quality.

### 2.2. Green Synthesis of Cu NPs

Cu NPs were made by heating a 0.1 g/mL solution of *L. japonica* extracts in a flask at 80 °C in a water bath while magnetically swirling the mixture. Poloxamer 407 and 100 mL of 0.1 M CuCl_2_ were then added dropwise, and the mixture was maintained at 80 °C and agitated for 8 h. After being centrifuged at 9000 rpm for 10 min to remove debris, the solution was dialyzed in a dialysis bag (MW: 300). The leftover colloidal solution was diluted and characterized for use in other studies.

Poloxamer 407 is a non-ionic surfactant known for its excellent stability and dispersibility. During the synthesis of Cu NPs, Poloxamer 407 adsorbs onto the surface of the nanoparticles, forming a protective layer. This layer prevents the aggregation of Cu NPs caused by their high surface energy, thereby ensuring their dispersion and stability.

### 2.3. Characterization of Cu NPs

The size, morphology, and dispersion of Cu NPs were examined using HRTEM (Tecnai G2 F30 S-TWIN, *FEI*, *USA*), Cu nanoparticles XRD (D8 ADVANCE, *Bruker-AXS*, *Germany*) investigation and crystallinity measurement. The molecular structure of *L. japonica* extracts and Cu NPs was determined utilizing FTIR (ALPHA, *BRUKER*, *USA*) spectroscopy. To determine the absorbance at various concentrations, a UV–Visible Spectrophotometer (Cary 60, *Agilent*, *USA*) was utilized, and the absorbance range was 0~3.

### 2.4. Catalytic Activity of Cu NPs

The dye solution was added to an NaBH_4_ solution at a specific concentration, serving as an excess reducing agent, with or without the addition of a Cu NP solution. Using ultrapure water as the baseline reference, the UV absorption spectra of three different dye solutions, with and without the Cu nanoparticle catalyst, were recorded using a UV–Vis spectrophotometer. The catalytic efficiency of the catalyst for different compounds was then analyzed. The reaction dosages for each sample are detailed in Table 1. All of the work here was performed at ambient temperatures [36].

### 2.5. Catalytic Activity of Cu NPs Modified Fiber Balls

Coated fiber balls were filled with Cu/*Lonicera japonica* Thunb composite powder obtained through freeze-drying to provide a simple simulated catalytic bed for the numerous reductions of methylene blue [37]. Methylene blue was reduced catalytically by NaBH_4_ to measure Cu/Lj-C’s catalytic activity. After each cycle, fixed amounts of reactants and reducing agents (1 g of Cu NPs, 50 mL of 3 mM MB, and 2 mL of 0.1 M NaBH_4_) were gradually added to the device for catalytic reduction. Upon completing each run, an equal volume of MB dye was replenished, and the next catalysis was performed under the same conditions as the initial reaction to evaluate the cyclic performance. All tests were performed at ambient temperatures [38].

### 2.6. Cytotoxicity of Cu NPs

HUVEC cells and MC3T3-E1 cells were cultured in Dulbecco’s modified Eagle medium (DMEM) at 37 °C and 5% CO_2_. HUVEC cells and MC3T3-E1 cells were obtained from the stem cell bank of the Chinese Academy of Sciences. The medium was added with 10% heat-inactivated fetal bovine serum (FBS) and 1% penicillin/streptomycin antibiotics and kept in a humid environment of 37 °C and 5% CO_2_. These cells were inoculated and cultured in 24-well plates at a cell density of 1 × 10^4^ cells/well in an incubator at 37 °C and 5% CO_2_ for one day. The 24-well plates were divided into six groups (blank control group, n = 6):0, 0.625, 1.25, 2.5, 5, and 10 μg/mL groups. The volume of culture medium used for each well plate was 2 mL. Cell viability was assessed using the CCK-8 kit and L/D vital dye, respectively [39]. The original medium was replaced after 24 and 48 h with 100 mL of DMEM containing 10 mL of CCK-8, and the media was cultivated at 37 °C for 2.5 h. The MultiskanFC enzyme labeling device evaluated the absorbance, and the OD 450 nm was recorded and displayed. A drug-loaded scaffold’s cell-inhibitory action might be more clearly shown using L/d reactive dye. The growth media was removed from the well plate after the cells had been cultured there for 24 and 48 h. Then, 100 mL of PBS was added, and the cells were treated there for 15 min with 4 mM calcein and 2 mM ethidium homodimer with dimer. Dead cells are depicted in red, whereas living cells are highlighted in green.

### 2.7. Antibiosis of Cu NPs

The technique of microbial inhibition zone on a solid porous gel plate was used to investigate the antibacterial ability of our Cu NPs. Four microbiological strains, representing Gram-positive bacteria, Gram-negative bacteria, yeast, and mycete, respectively, were employed in the test: *Escherichia coli* (ATCC 25922), *Staphylococcus aureus* (ATCC 25923), *Candida albicans*, and *Aspergillus niger*. Cu NPs samples 1 and 2 were made using the same synthesis process but at different concentrations. The following tests were carried out using *E. coli* and *S. aureus*. First, 12 h were spent shaking bacteria suspension solutions, including *E. coli* and *S. aureus*, in a 37 °C incubator. Then, a double-layer agar media plate was created using the following steps: spread 20 mL of molten LB agar media onto a 90 mm Petri dish as the down-layer; after cooling, add a specific volume of bacteria suspension solution to LB agar media at a temperature of roughly 50 °C, shake the media for an even mixing, then spread this media onto the down-layer. In such an up-layer medium, there were around 108–109 colony-forming units (CFU) per milliliter of fluid. We made a suspension solution for *C. albicans* by shaking it for 12 h at 28 °C. Then, a 300 mL solution containing 30 mL of modified Martin medium that had been autoclaved and cooled to 45 °C was added. Onto a 90 mm Petri plate, the mixture was dispersed after being evenly mixed.

The aseptic filter paper discs were placed on top of the plates after being impregnated with the Cu NPs, the extract solution, and the PBS buffer (used as a control). The microbe inhibition zones were identified after incubating the plates for 24 h at 37 °C (*E. coli* and *S. aureus*) or 28 °C (*C. albicans*).

*Aspergillus Niger* medium was made by adding 1 mL of Cu NPs sample, extract, or PBS buffer to 30 mL of modified Martin medium. Some Bacillus melanosporum spores were placed in a medium dish after the medium had been cooled, and the dish was then incubated at 28 °C. Mycelium growth was seen after 24 and 48 h.

## 3. Results and Discussion

### 3.1. HR-TEM Analysis of Cu NPs

**Figure 1:**
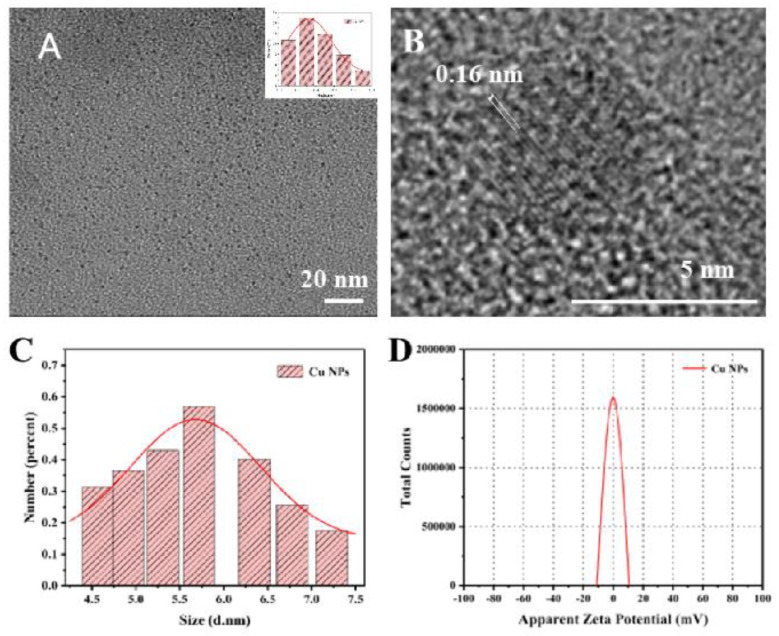
(**A**,**B**) HRTEM images of Cu NPs at different magnifications; (**C**) dynamic light scattering particle size analyzer micrograph, and (**D**) zeta potential analysis graph of green synthesized Cu nanoparticles by using *Lonicera japonica* Thunb extract.

The synthesized Cu NPs, with an average particle size of 2–4 nm and uniform dispersion, are shown in Figure 1A. Due to their small particle size, they exhibit a high specific surface area [40,41]. The Cu NPs also display a lattice surface spacing of 0.16 nm, as observed in Figure 1B.

### 3.2. DLS Analysis of Cu NPs

The average particle size of the synthesized Cu NPs, as shown in Figure 1C, is 6 ± 1 nm, which is slightly larger than the size determined by TEM due to the absorption of a layer of plant-derived molecules on the surface of the Cu NPs. The zeta potential is measured to be −0.087 mV, within the range of −10 mV to +10 mV, as depicted in Figure 1D. When the absolute value of the zeta potential of Cu NPs in an aqueous solution is less than approximately ±30 mV, the system may become unstable and prone to aggregation. In this study, the measured zeta potential of the nano-copper falls within the range of −10 to +10 mV. This low zeta potential indicates that the repulsive forces between particles are insufficient to counteract attractive forces, such as van der Waals forces, potentially leading to particle aggregation.

### 3.3. XRD Characterization of the Biosynthesized Cu NPs

**Figure 2:**
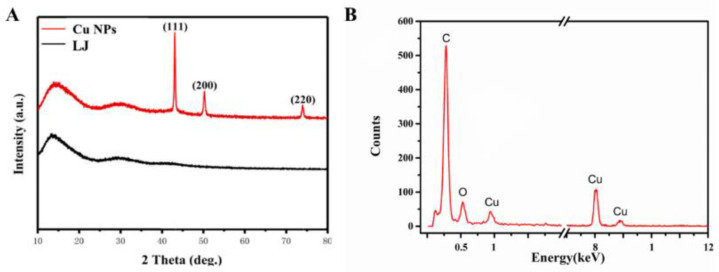
(**A**) XRD pattern of *L. japonica* and Cu NPs (**B**) EDS pattern of Cu NPs.

The XRD pattern of Cu NPs, shown in Figure 2A, reveals peaks that are consistent with those of pure copper. The diffraction peaks at 2θ values of 43°, 50°, and 74° correspond to the (111), (200), and (220) planes, respectively, indicating that the sample matches the characteristics of pure copper [10]. The XRD pattern, along with previous EDS analysis, confirms the formation of Cu(0) nanoparticles [42]. The peaks in the EDS spectrum of Cu NPs, rich in carbon and oxygen, can confirm various chemical compounds on the surface of Cu NPs from the source of *Lonicera japonica* Thunb aqueous extract [43].

### 3.4. SEM Analysis of Cu/LJ-C

**Figure 3:**
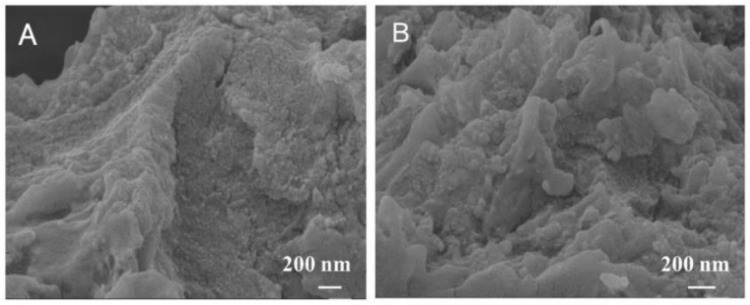
The SEM image Cu/*Lonicera japonica* Thunb nanocomposite (**A**,**B**).

**Figure 4:**
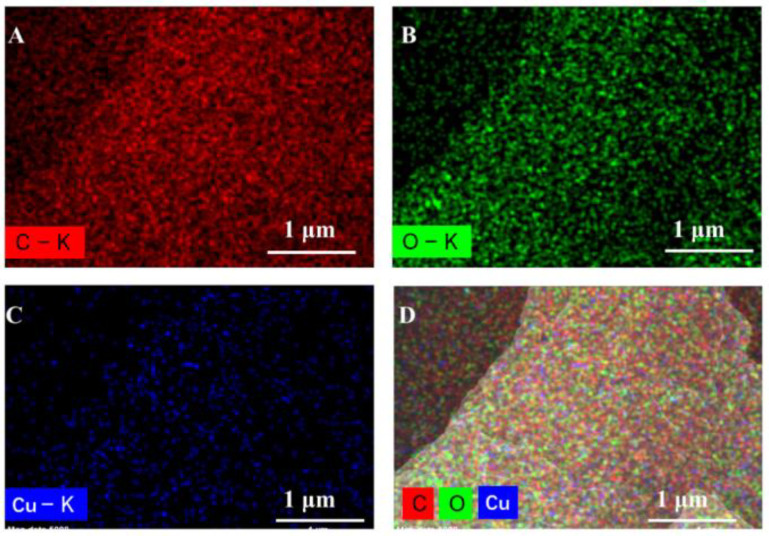
The SEM image Cu/*Lonicera japonica* Thunb nanocomposite element mapping (**A**–**D**).

The plant cellulose network in the powdered *Lonicera japonica* Thunb provides a suitable environment for the loading of Cu NPs, facilitated by the release of internal components during the synthesis of Cu NPs from the reductive compounds of *Lonicera japonica* Thunb. To address the challenge of recovering copper nanoparticles, we designed an experiment in which copper nanoparticles were synthesized and loaded in a single step using the aqueous extract of *Lonicera japonica* Thunb. The Cu NPs attached to the cellulose network were obtained by centrifugation and dialysis. The results in Figure 3A,B clearly show that numerous Cu NPs adhere to the plant cellulose of *Lonicera japonica* Thunb powder. The Cu mapping signal in Figure 4A–D confirms that Cu has been effectively incorporated into the plant cellulose network of *Lonicera japonica* Thunb powder.

### 3.5. A Probable Mechanistic Pathway for the Synthesis of Cu NPs

**Figure 5:**
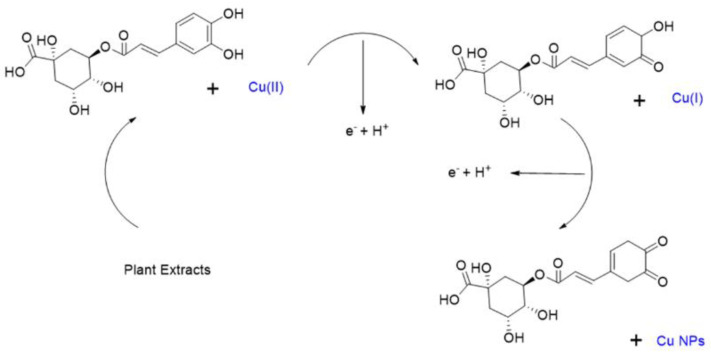
Possible mechanism of transforming copper salt to copper nanoparticles.

In the synthesis process, reducers and stabilizers such as flavonoids and phenolic compounds are employed. The copper ions are eventually broken down into copper atoms, and when the atoms self-assemble, new crystal nuclei of copper nanoparticles are created. In addition to playing a crucial part in the reduction of Cu^2+^ to Cu NPs, flavonoids can directly scavenge ROS and donate electrons or hydrogen atoms. To keep the copper nanoparticles stable, *Lonicera japonica* Thunb has a coating of polysaccharides and alkaloids surrounding them (Figure 5) [33,44].

### 3.6. FT-IR Characterization of Lonicera japonica Thunb Extract and Cu NPs

**Figure 6:**
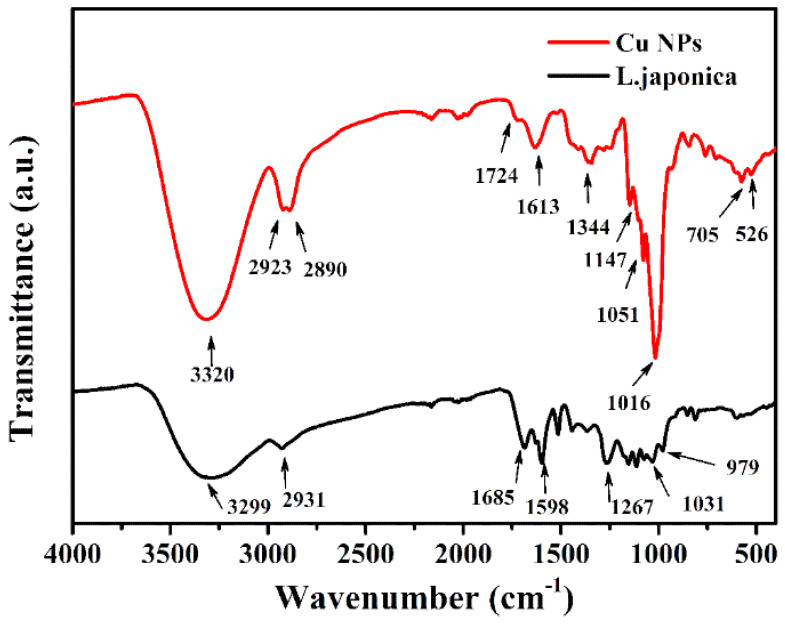
FT-IR spectra of *Lonicera japonica* Thunb and Cu NPs.

As shown in Figure 6, the observed bands at 3320 cm^−1^ and 2923 cm^−1^ correspond to the N-H stretching of amines and the C-H stretching of the alkyl group, respectively. The peaks at 2890 cm^−1^ and 1613 cm^−1^ represent the O-H stretching of the carboxylic acid and the N-H bending of the amine, respectively [45]. The spectrum also contains a faint band at 526 cm^−1^ that is generated by Cu-O tensile vibration. Other absorption bands at 2890 cm^−1^ and 1344 cm^−1^ demonstrated the presence of bioorganic compounds on the surface of Cu NPs. The majority of organic compounds on the surface of Cu nanoparticles are extract residues that coordinate with Cu^2+^ during reduction. The combination of these secondary metabolites with the surface of Cu NPS stabilizes Cu NPs and prevents their aggregation in aqueous solutions.

### 3.7. Dyes Reduction Catalyzed by Cu NPs

**Figure 7:**
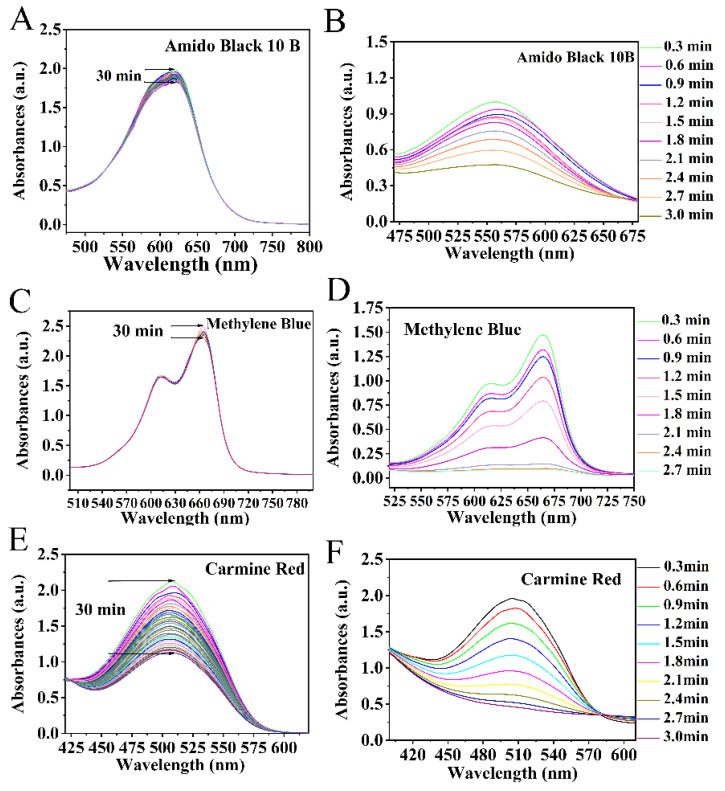
UV–Vis absorption of AB-10B versus time with (**A**) and without (**B**) Cu NPs; UV–Vis absorption of MB versus time with (**C**) and without (**D**) Cu NPs; UV–Vis absorption of CR versus time with (**E**) and without (**F**) Cu NPs.

In the presence of a reductant, the catalytic reduction of amino black 10B (AB-10B) can be monitored using a UV–Vis spectrophotometer. Without Cu-NPs as a catalyst, the AB-10B reduction rate was rather sluggish. After half an hour, the absorbance of the solution decreases by approximately 7.60% (Figure 7A). However, when copper nanoparticles are added, the absorbance of the solution decreases by 50 percent within three minutes (Figure 7B). The primary peak varies between 602 and 542 nm. The conjugated system and the diazo bond of the amino black dye are simultaneously annihilated.

The outstanding catalytic activity of the prepared Cu NPs is further demonstrated by the catalytic reaction between MB and CR. Without Cu nanoparticles, the reaction rate of methylene blue with NaBH_4_ was extremely sluggish, the absorbance change was comparable to that of AB-10B, and MB barely reacted with NaBH_4_. When Cu NPs were added to the MB solution, the intensity of absorption at 665 nm decreased swiftly with time (Figure 7C). The catalytic degradation can be finished in 2.7 min (Figure 7D). Without Cu NPs as a catalyst, the rate of CR reduction was relatively sluggish, and the absorbance of the solution decreased by approximately 47.1% after 0.5 h (Figure 7E). When copper nanoparticles are introduced, the reaction rate changes considerably, and the absorbance of the solution drops by 76.5 percent within three minutes (Figure 7F).

**Figure 8:**
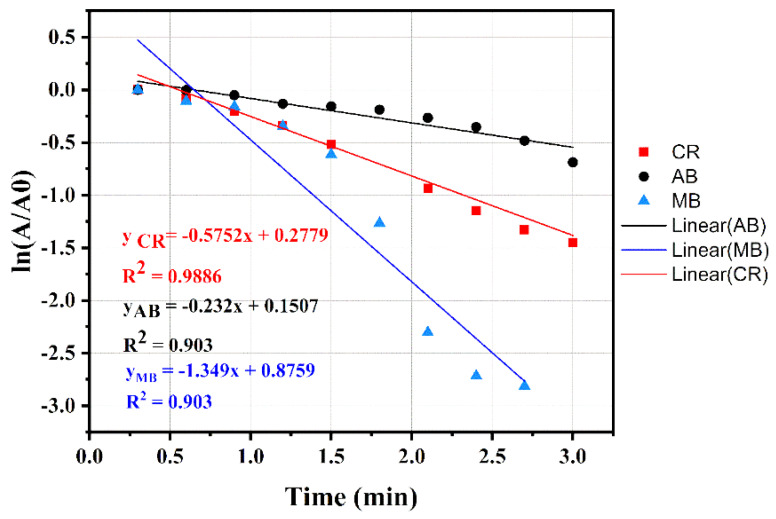
The variation of ln(A/A_0_) versus time and linear equation for the reduction of AB-10B, MB, and CR.

The ability of the produced Cu NPs to quickly degrade AB-10B, MB, and CR shows that they are effective dye degradation catalysts with a wide application range (Figure 8). The computed apparent rate constants (kapp) were 2.32 × 10^−1^ min^−1^, 1.349 min^−1^, and 5.752 × 10^−1^ min^−1^ for the three processes: AB-10B, MB, and CR.

During the reaction, the concentration of NaBH_4_ could be termed constant because it was present in excessive quantities. Then, it could be supposed that the reduction of AB-10B, MB, and CR follows pseudo-first-order kinetics [13]. Following is the kinetic equation for this catalytic reaction:

ln(C_t_/C_0_) = ln(A_t_/A_0_) = −kt (1)

k is the rate constant, C_t_ and C_0_ are the organic dye concentrations at time t, and the beginning state (t = 0), and t is the time variable. A_t_ and A_0_ are the absorbance of organic dyes at time t and the beginning state (t = 0), and t is the time variable.

**Figure 9:**
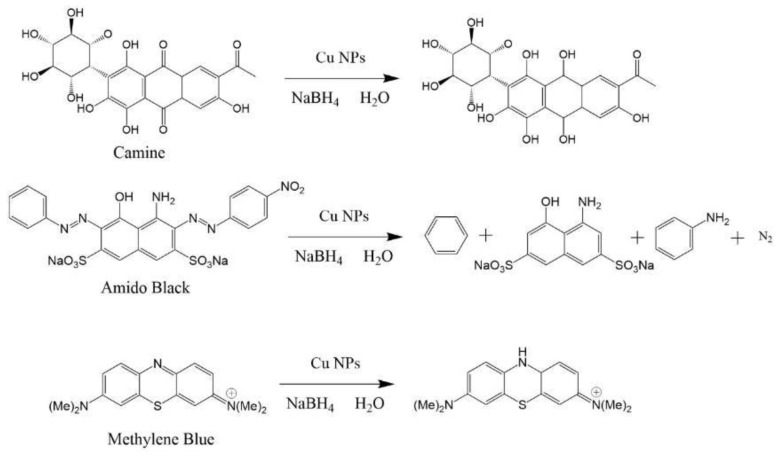
The reaction scheme for reducing AB-10B, MB, and CR using Cu NPs.

Nano-metal catalysts often exhibit a greater catalytic effect than conventional catalysts in many processes due to their high specific surface area and surface energy, which provide more active sites. As shown in Figure 9, copper nanoparticles facilitate electron transfer from NaBH_4_ ions to organic dyes, enabling a rapid catalytic reduction of the dyes. Comparing our findings with similar studies (see Table 2), we found that the nano-copper catalyst synthesized using *Lonicera japonica* extract as a starting material demonstrated the highest catalytic efficacy. This superior performance is likely due to the green synthesis method employed, which yielded nano-copper catalysts with minimal particle size.

### 3.8. Dyes Reduction Catalyzed of Cu NPs Cu-Modified Fiber Balls

**Figure 10:**
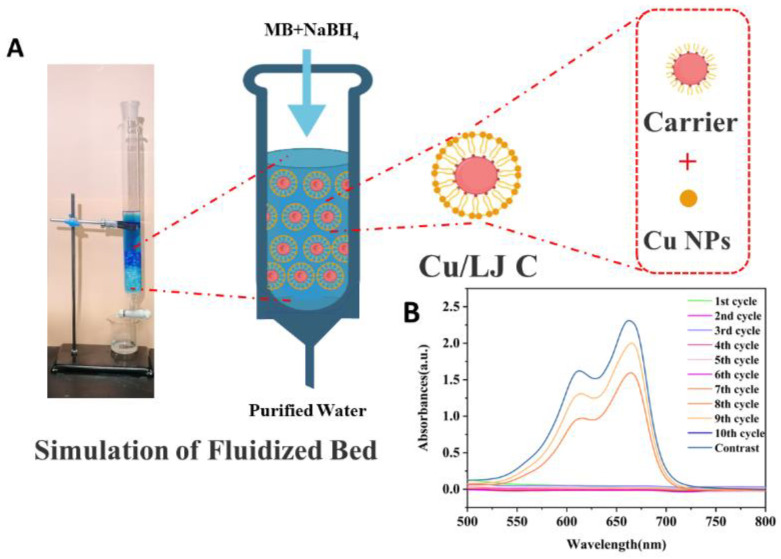
(**A**) The experimental process of catalysis of methylene blue in a fluidized bed simulation; (**B**) the reusability of Cu/LJ-C for the reduction of MB by NaBH_4_.

Reusability and recoverability are essential for evaluating the effectiveness of catalyst beds in industrial applications for catalytic degradation. In this study, we performed experiments to assess the reusability of the porous Cu/Lj-C catalyst bed for repeated dye degradation cycles. Figure 10A illustrates the experimental setup for the multi-cycle catalytic degradation of MB dye, using a consistent amount of catalyst and reactants throughout. After each catalytic cycle, the Cu/Lj-C catalyst was thoroughly washed with distilled water, removing adsorbed products under identical conditions as in previous tests. Figure 10B shows the time required for complete MB degradation in each cycle. Although continuous cycling slightly prolonged the reaction time, the dye was entirely degraded after the first cycle through to the seventh, indicating consistent catalytic performance. Interestingly, catalytic efficiency increased up to the tenth cycle, though performance slightly declined in subsequent cycles due to material loss during washing and potential blockage of active sites by reaction intermediates. Figure 3A presents the SEM image of Cu NPs before cycling, while Figure 3B shows the SEM image of Cu NPs after seven cycles. The images reveal that Cu NPs, without cycling, retain a smaller particle size. However, after multiple cycles, the particle size of the Cu NPs increases significantly, and their quantity decreases. This change likely contributes to the slower reduction rate of catalytic dyes. These findings demonstrate that the Cu/Lj-C catalyst maintains effective catalytic activity over seven high-frequency cycles, highlighting its strong reusability for dye degradation applications.

### 3.9. Cytotoxicity of Cu NPs

**Figure 11:**
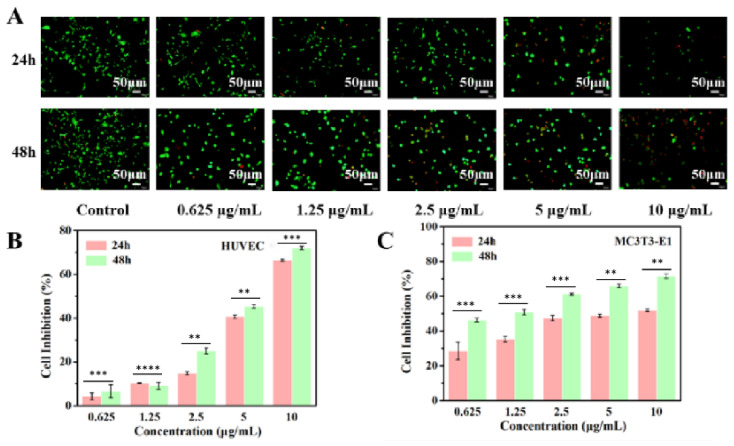
(**A**) Fluorescence stain of MC3T3-E1 cells with different concentrations of nano-copper at 24 h and 48 h; (**B**) cytotoxicity of nano-copper at different concentrations for 24 h and 48 h with HUVEC; and (**C**) cytotoxicity of nano-copper at different concentrations for 24 h and 48 h with MC3T3-E1 cell. (** *p* < 0.01, *** *p* < 0.001, **** *p* < 0.0001).

Small, spherical copper nanoparticles can enter cells and induce cytotoxicity through various mechanisms. They can elevate ROS levels beyond a cell’s protective threshold, leading to lipid peroxidation, membrane damage, and the activation of apoptotic pathways and endoplasmic reticulum stress [42]. CCK-8 and live/dead (L/D) staining assays were conducted to assess the cytotoxicity of copper nanoparticles, with the results shown in Figure 11. The median lethal dose (LD_50_) was determined to be 37.64 µg/mL, with HUVEC cells showing a 1.04 ± 0.06% greater inhibitory effect after 48 h than at 24 h, suggesting that toxicity increased with concentration and exposure time. For MC3T3-E1 cells, copper nanoparticles showed a 1.41 ± 0.21% greater inhibitory effect at 48 h than at 24 h, with an LD_50_ of 7.50 µg/mL, indicating a more pronounced toxic effect on these cells as copper nanoparticle concentration increased. Live/dead (L/D) staining assays reveal that Cu NPs exhibit toxicity toward normal cells. Using MC3T3-E1 cells as an example, the cell inhibition rate increases with both exposure time and Cu NP concentration. Previous studies [43,44,45] have reported that particles with diameters of 40–60 nm exhibit higher cytotoxicity compared to smaller (25 nm) and larger (60–80 nm) particles. This size-specific toxicity is likely attributed to the smaller hydrodynamic radius of particles within the 40–60 nm range in cell culture medium, which enhances cellular uptake and ultimately results in more severe cytotoxicity.

Currently, numerous studies have reported on the toxicity of Cu NPs. The mechanisms underlying their toxic effects on organism growth and metabolism are generally attributed to two main aspects: (1) Cu NPs induce the production of reactive oxygen species (ROS) within organisms, leading to oxidative stress, and (2) the release of Cu^2+^ ions from Cu NPs results in toxic effects [46]. The presence of Cu^2+^ ions can enhance the Fenton reaction, generating large amounts of hydroxyl radicals that degrade essential biomolecules (lipids, proteins, nucleic acids), ultimately leading to cell destruction [47]. Additionally, the size and shape of Cu NP particles are critical factors contributing to cellular oxidative damage. Copper nanoparticles can oxidize certain organic macromolecules, producing ROS that impact mitochondrial apoptosis, protein denaturation, and DNA damage, culminating in cell destruction [48].

The experimental results indicate that toxicity increases with prolonged exposure time, aligning with the mechanism by which copper nanoparticles (Cu NPs) induce reactive oxygen species (ROS) generation, leading to oxidative stress. As exposure time extends, intracellular ROS levels may rise beyond the cell’s protective capacity, triggering oxidative stress reactions and ultimately resulting in cytotoxicity. Additionally, the results demonstrate that higher concentrations of Cu NPs have a more pronounced toxic effect on cells, likely due to the increased release of Cu^2^⁺ ions. Elevated concentrations of Cu NPs may release more Cu^2^⁺ ions, intensifying the Fenton reaction and causing greater cell damage.

### 3.10. Antibiosis of Cu NPs

**Figure 12:**
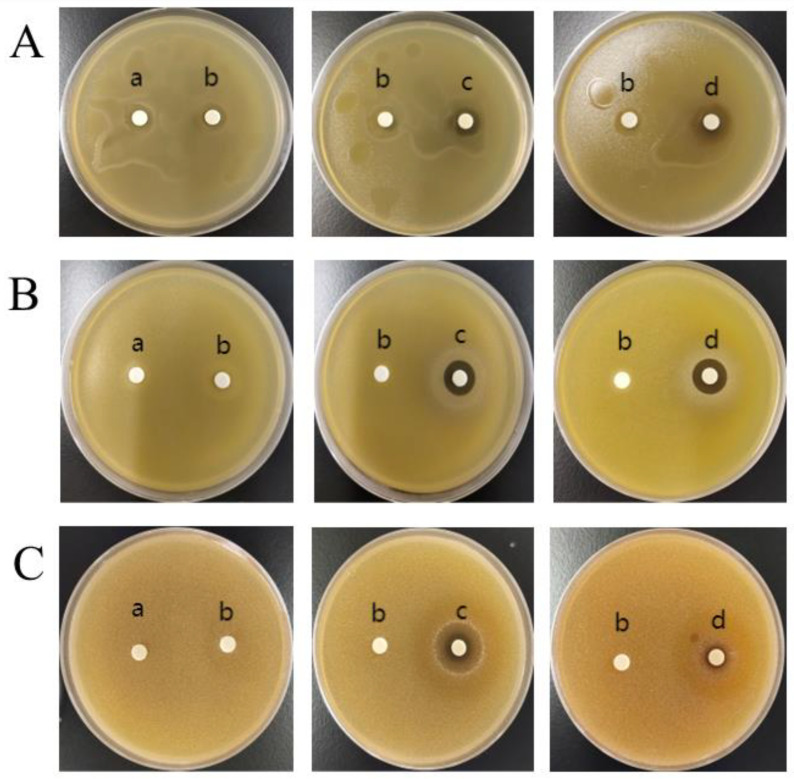
Cu NPs inhibition zones on (**A**) *E. coli*, (**B**) *Staphylococcus aureus*, and (**C**) *Candida albicans* plates. For all photographs, a represents the blank control group in PBS, b represents the sample of *Lonicera japonica* Thunb extract, c represents the sample of 10 μg/mL Cu NPs, and d represents the sample of 20 μg/mL Cu NPs.

**Figure 13:**
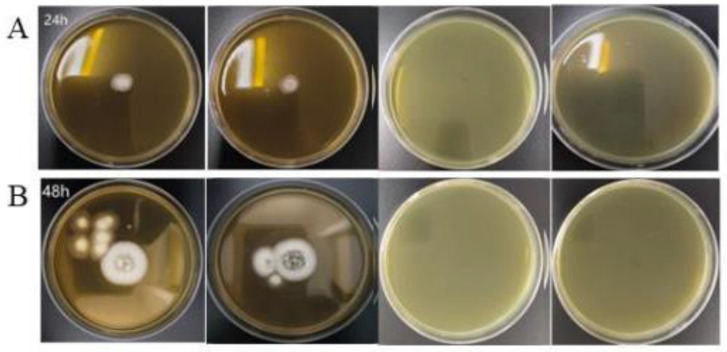
Growth inhibition of Cu NPs samples to *Aspergillus niger*: (**A**) 24 h and (**B**) 48 h. The media of plates from left to right were modified Martin medium mixed with 1 mL of PBS buffer, the extract solution without copper, and Cu NPs samples, respectively.

The results of the microbial inhibition assay for Cu NPs are presented in Figure 12 and Figure 13. Figure 12 illustrates the antimicrobial effects of Cu NPs on *E. coli*, *S. aureus*, and *C. albicans*. Both Cu NP samples showed inhibitory activity against all three strains, with inhibition being more pronounced for *S. aureus*, suggesting that Gram-positive bacteria are more sensitive to these Cu NPs. Previous studies have indicated that green-synthesized copper nanoparticles can vary in antibacterial efficacy across bacterial types [49,50]. Some studies found higher efficacy against Gram-positive bacteria, while others reported the opposite, likely due to differences in synthesis methods.

For *C. albicans*, Cu NPs exhibited inhibitory activity, which aligns with the established inhibition mechanisms of copper against yeast. After 24 and 48 h of incubation, no growth of *Aspergillus niger* was observed on media containing Cu NPs (Figure 13), indicating the excellent antifungal properties of these Cu NP samples. In contrast, the extract solution without copper showed no antimicrobial activity against any of the four tested strains.

Copper is an essential trace element in the human body, vital for hemoglobin synthesis, involvement in hematopoiesis, and supporting metabolic functions in biological tissues [35]. Copper nanoparticles (Cu NPs) exhibit strong bactericidal properties, with robust adsorption and antimicrobial activity, and possess a broad spectrum of long-lasting antibacterial, antifungal, and anti-mildew effects [51]. Research into the antibacterial mechanisms of Cu NPs is increasing, with several prevailing theories: (1). Membrane Binding and Disruption: Cu^2+^ ions bind to bacterial cell membranes and proteins, damaging the cell wall, disrupting metabolic processes, and ultimately causing cell death [13,52] (2). Membrane and Wall Degradation: Cu NP particles disrupt cell walls and membranes, leading to leakage of intracellular components, which blocks biochemical metabolism and results in bacterial death. (3). ROS Production and Oxidative Damage: Cu NPs promote the production of reactive oxygen species (ROS), oxidizing cell structures, degrading organic macromolecules, and damaging DNA, leading to cell death. Mechanisms (1) and (2) are extracellular, involving nanoparticle-induced membrane perforation and disruption of membrane stability. Mechanism (3) is intracellular, achieving antibacterial effects through interactions with biological macromolecules and oxidative damage.

Gram-positive bacteria have a thicker peptidoglycan cell wall structure, greater rigidity, and lack an outer membrane compared to Gram-negative bacteria. Consequently, Gram-positive bacteria are generally less susceptible to cell wall damage caused by the “nano-perforation” effect of Cu NPs than Gram-negative bacteria. However, in this study, the antibacterial experiments revealed that Cu NPs exhibited a more pronounced inhibitory effect on Gram-positive bacteria, suggesting that intracellular antibacterial action is likely the dominant mechanism here. The *Lonicera japonica* extract, used as a capping agent, coats the surface of the Cu NPs, influencing their size, shape, and surface characteristics, which likely reduces the “nano-perforation” effect. This supports the idea that extracellular mechanisms are not the primary mode of action for Cu NPs in this study. Additionally, the quantum properties of Cu NPs can lead to reactive oxygen species (ROS) generation. In the green synthesis system for Cu NPs, carbon quantum dots can enhance electron transfer and facilitate redox reactions, contributing to the bacteriostatic and bactericidal effects of the nanoparticles. The results of the antibacterial experiment show that as the concentration of the Cu NP solution increases, accompanied by a corresponding rise in carbon quantum dots, the antibacterial effect is significantly enhanced, supporting this perspective.

## 4. Conclusions

In this study, a simple and environmentally friendly method was developed to produce nano-sized copper using *Lonicera japonica* water extract as both a reducing and stabilizing agent. The resulting copper nanoparticles, with a uniform particle size of 2–4 nm, were successfully synthesized, as confirmed by HRTEM, SEM, DLS, EDS, and XRD analyses. These nanoparticles demonstrated excellent catalytic reduction activity for the dyes MB, AB-10B, and CR. The apparent rate constants (kapp) for AB-10B, MB, and CR are 2.32 × 10^−1^ min^−1^, 1.349 min^−1^, and 5.752 × 10^−1^ min^−1^, respectively. A simple Cu/Lj-C catalytic bed was established for multi-dye decolorization, with the dyes recovered via centrifugation. Experimental results showed that the Cu/Lj-C catalytic bed maintained strong catalytic activity over seven cycles. Cytotoxicity assays indicated that the green-synthesized copper nanoparticles from Lonicerae japonica extract were cytotoxic to HUVEC and MC3T3-E1 cells, with median lethal doses of 37.64 μg/mL and 7.50 μg/mL, respectively. Additionally, microbial inhibition tests demonstrated the copper nanoparticles’ strong antibacterial activity, effectively inhibiting the growth of four different microorganism strains. These findings highlight the catalytic, antibacterial, and other functional properties of nano-copper synthesized from *Lonicera japonica* extract, highlighting its potential for significant advancements in removing organic dyes from industrial wastewater. By studying cytotoxicity, we can gain deeper insights into the effects of copper nanoparticles (Cu NPs) on cells, providing a scientific basis for their safe application in various fields. Additionally, given the strong antibacterial properties of Cu NPs, cytotoxicity research supports the development of novel antibacterial materials with enhanced efficiency and reduced toxicity.

## Figures and Tables

**Table 1:** Concentration and volumes of dyes used in the experiments.

Dyes/NPs	C_dyes_	V_dyes_	C_NaBH4_	V_NaBH4_	C_Cu NPs_	V_Cu NPs_
**AB-10B (Amido Black 10B)**	2 mM	1 mL	0.1 M	2 mL	30 μg/mL	0.2 mL
**MB (Methylene Blue)**	2 mM	1mL	0.1 M	2 mL	30 μg/mL	0.2 mL
**CR (Carmine Red)**	2 mM	1 mL	0.1 M	2 mL	30 μg/mL	0.2 mL

**Table 2:** Comparison of the catalytic reduction performance of nano-copper catalysts for organic dyes.

Dyes	Biogenic Source	Nano	Kapp	
AB	*Lonicera japonica* Thunb	Cu NPs	2.32 × 10^−1^ min^−1^	This work
AB	Green coffee bean	Cu NPs	1.722 × 10^−2^ min^−1^	32
MB	*Lonicera japonica* Thunb	Cu NPs	1.349 min^−1^	This work
MB	Phragmanthera austroarabica	Cu NPs	3.276 × 10^−2^ min^−1^	45
MB	Green coffee bean	Cu NPs	4.458 × 10^−1^ min^−1^	32
CR	Phragmanthera austroarabica	Cu NPs	4.829 × 10^−2^ min^−1^	45
CR	*Lonicera japonica* Thunb	Cu NPs	5.752 × 10^−1^ min^−1^	This work

## Data Availability

The raw data supporting the conclusions of this article will be made available by the authors on request.
